# Reconstruction with fibular osteocutaneous free flap in patients with mandibular osteoradionecrosis

**DOI:** 10.1186/s40902-015-0007-3

**Published:** 2015-02-05

**Authors:** Min Gyun Kim, Seung Tae Lee, Joo Yong Park, Sung Weon Choi

**Affiliations:** Oral Oncology Clinic, Research Institute and Hospital, National Cancer Center, Il san east, Il san road 323, 2nd floor, Goyang, South Korea

**Keywords:** Osteoradionecrosis, Mandible, Fibular, Radiation, Free flap

## Abstract

**Background:**

Osteoradionecrosis is a delayed complication from radiation therapy which causes chronic pain, infection and constant deformity after necrosis. Most of the osteoradionecrosis occurs spontaneously or after the primary oncologic surgery, dental extraction or by trauma of prosthesis. The treatment of osteoradionecrosis relies on both conservative measures and surgical measures. The fibular osteocutaneous free flap has become more popular choice for reconstruction of maxillofacial defects as a treatment of osteoradionecrosis.

**Methods:**

We presented our experiences from 7 patients with osteoradionecrosis who have had reconstruction surgery with fibular osteocutaneous free flap at National Cancer Center during the recent 5 years. We performed segmental mandibular resection with fibular osteocutaneous free flap for all 7 patients of advanced osteoradionecrosis who were not controlled by conservative treatment such as wound irrigation, debridement, and antibiotics.

**Results:**

A wide range of techniques were available for the reconstruction of composite defects resulted from the treatment of advanced mandibular osteoradionecrosis. Significant improvement was noted in relieving pain and treating trismus after the surgery however difficulty in swallowing and xerostomia showed less improvement.

**Conclusions:**

We concluded that fibular osteocutaneous free flap can be performed safely in patients with osteoradionecrosis and yields positive outcomes with significantly increased success rate. The fibular osteocutaneous free flap was our preferred choice for the mandibular reconstruction due to its versatility and predictability.

## Background

In the oral and maxillofacial region, surgery and radiation therapy are the primary treatments for malignant tumors. Recent technological improvements in radiation therapy have resulted in a significant reduction of severe complications however pain, xerostomia, radiation caries and osteoradionecrosis are still serious complications remaining. Osteoradionecrosis causes serious aesthetic problem and oral malfunction which significantly reduces quality of life. Osteoradionecrosis is defined as response of impaired bone healing of irradiated bone tissue area due to poor blood circulation and lack of vitality for more than 3 months [1,2]. Osteoradionecrosis occurs most commonly in the mandible where blood circulation is maintained from the periosteum and in the end-artery system by inferior alveolar artery and vein. In general, osteoradionecrosis has been reported to occur within three years shortly after the radiation exposure. Osteoradionecrosis can occur spontaneously after the irradiation, after the tooth extraction, or through trauma by dentures and surgery. Also, jaw fracture with infected area and chronic pain lead to permanent deformity. The risk factors of osteoradionecrosis are the high doses of radiation in 6000-7000cGY and the frequent exposure to radiation due to short time interval which affect the deterioration of bone tissue directly. A tooth extraction before or after the surgery often serves a trigger point in developing osteoradionecrosis. In addition, alcohol consumption, tobacco use and improper practice of oral hygiene have also been reported as risk factors [3,4]. There is a hyperbaric oxygen therapy, antibiotics, irrigation and debridement as the conservative treatment of osteoradionecrosis. However, reconstructive surgery and radical jaw resection is necessary if conservative treatment fails. Mandible is a major component of the oral and maxillofacial region containing the teeth. Fibular free flap is a well known reconstruction approach for restoring proper oral function. Fibula free flap is known to be a safe and reliable method to get a sufficient height and appropriate thickness of corresponding mandible [5,6]. We presented our experiences and knowledge from 7 patients with osteoradionecrosis who have had reconstruction surgery with fibular osteocutaneous free flap at National Cancer Center during the recent 5 years.

## Methods

Fibular free flap is required for patients who have wide exposed and necrotic bone with severe pain, infection resulting in patholgic fracture, extra-oral fisula or osteolysis extending to the mandible border. We reviewed patients who had no improvement in symptoms after the conservative treatment and were treated for mandibular osteoradionecrosis by reconstruction with the fibular osteocutaneous free flap from July 2009 to July 2013 at National Cancer Center. Radiation image and medical records of patients were used to examine the outcomes. This research was conducted in accordance with the Helsinki Declaration.

## Result

**Figure 1:**
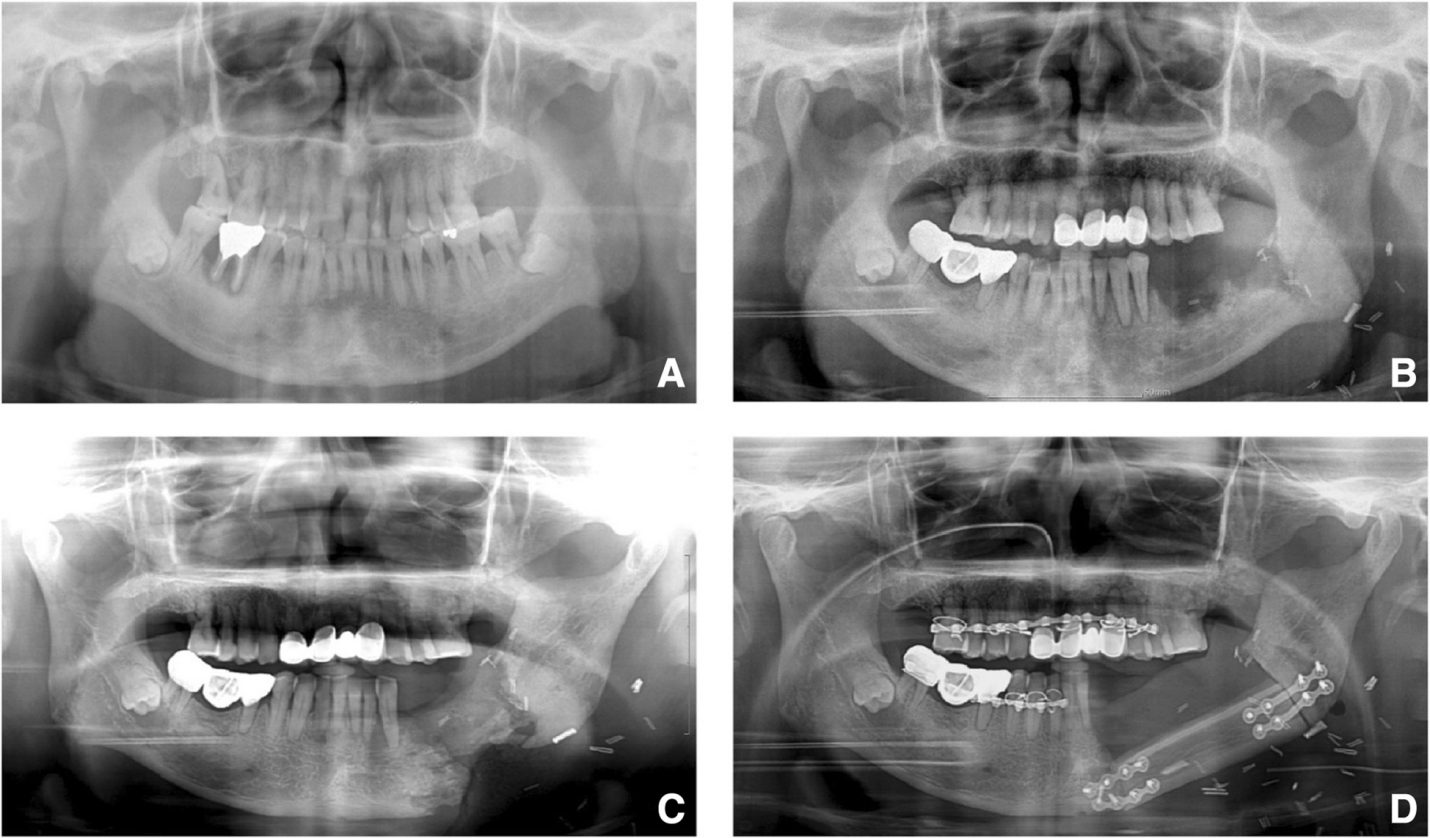
**Panoramic view of the osteoradionecrosis progression. (A)** Preoperative panoramic view of patient who extracted left 3^rd^ molar 5 years before the operation. **(B)** Panoramic view after 3 years from left 3^rd^ molar extraction. **(C)** Left mandible angle fractured after 5 years from extraction. **(D)** Postoperative panoramic view.

**Figure 2:**
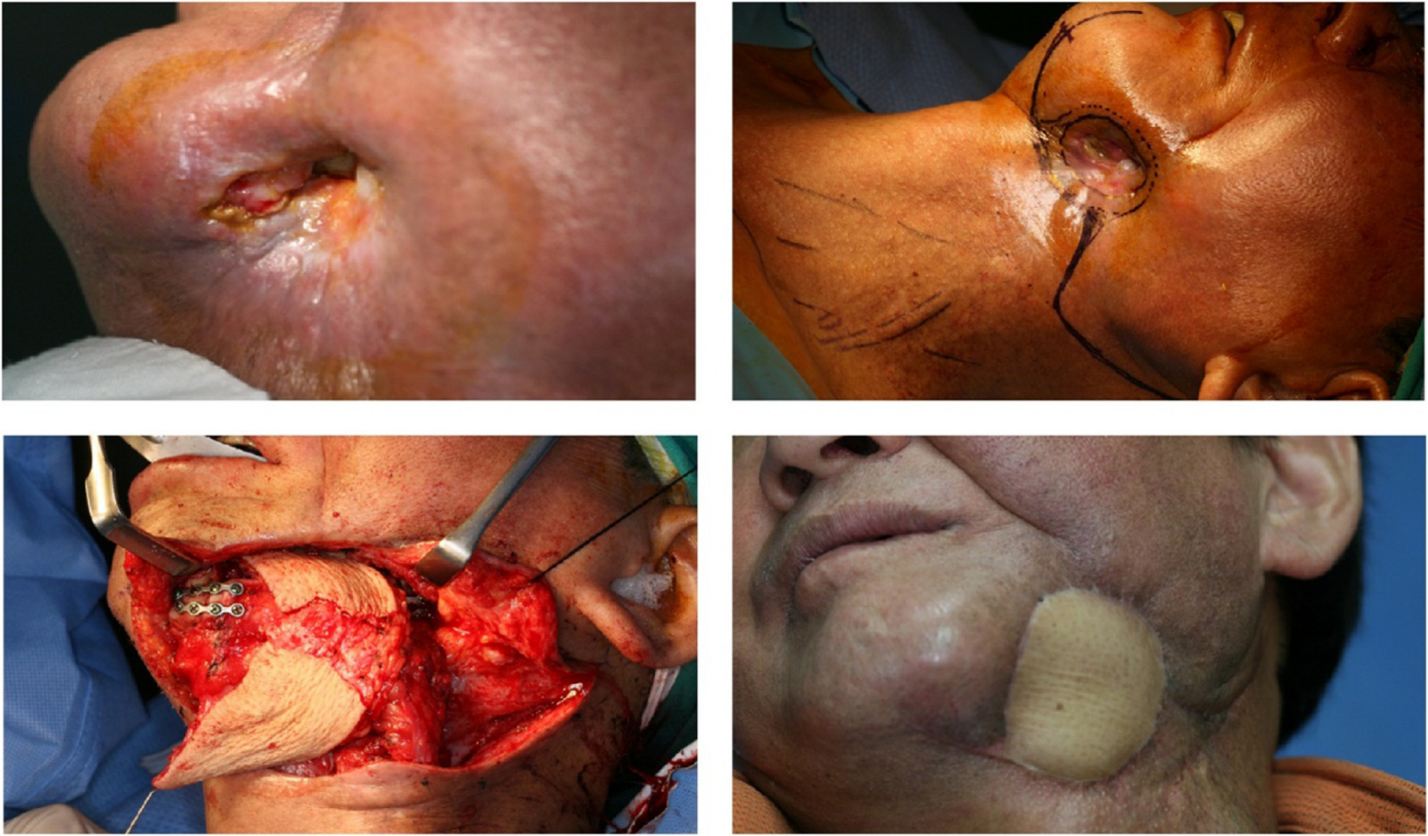
**Aggressive osteoradionecrosis formed extraoral fistula and saliva drained by fistula.** Soft tissue reconstruction with fistulectomy was required.

**Figure 3:**
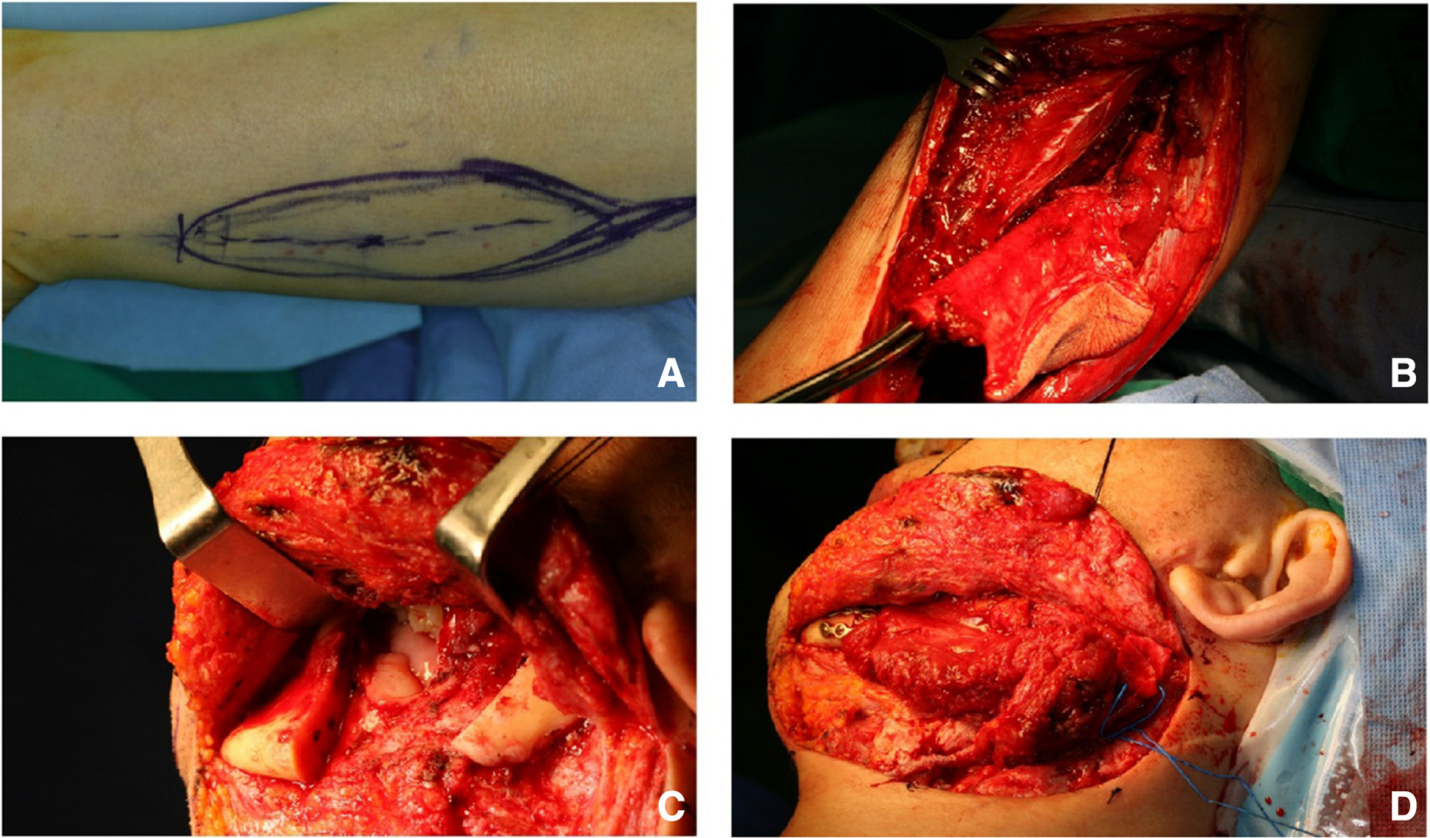
**Clinical view of the fibular free flap reconstruction. (A)** Initial clinical view **(B)** Fibular bone and soft tissue was harvested. **(C)** Necrotic mandible body was resected by pull through approach. **(D)** Vessels anastomosis was done.

**Figure 4:**
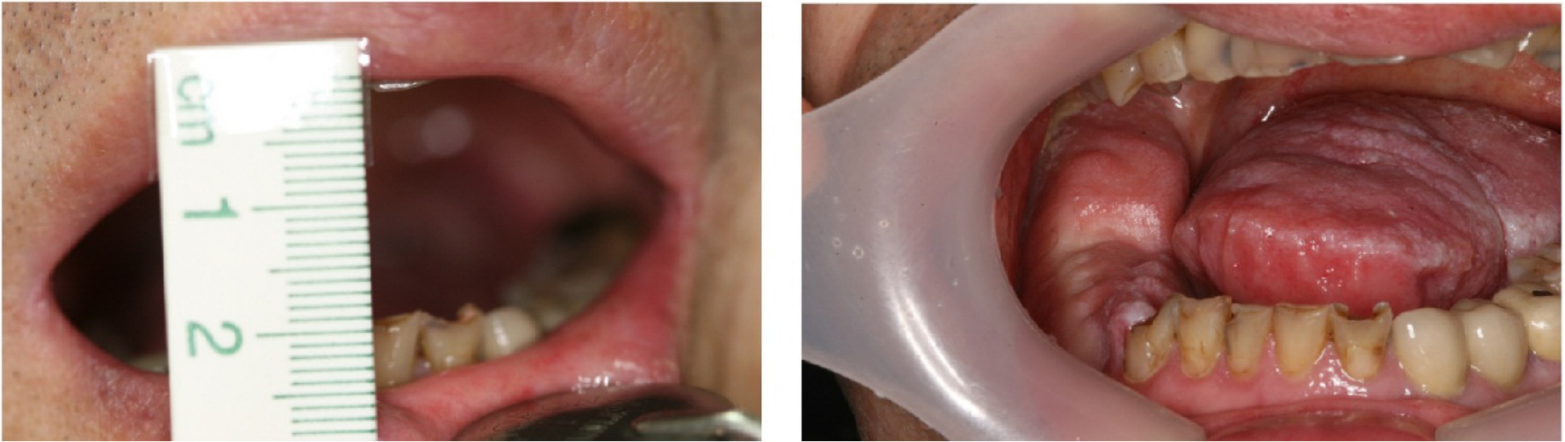
**Improvement of maximum mouth opening after the surgery.** (preoperative mouth opening limitation in the left and postoperative mouth opening in the right).

A total of 7 patients were studied; five were male and two were female. The patients were aged between 47 and 64 years of age (mean age 55 years). 6 cases were from the primary lesions of oral region; 2 cases from tongue, 1 from lips, 2 from gingiva, 1 from submandibuar gland. Only 1 chordoma case was from infratemporal region. The histopathologic diagnosis of 5 cases was squamous cell carcinoma except the mucoepidermoid carcinoma of submandibular glands and chordoma of infratemporal fossa (Table 1). The time to develop osteoradionecrosis following radiotherapy varies widely with an average of 3 years and 8 month; one case occurred within the 1 year, three cases occurred in 2 to 3 years, two cases occurred in 5 to 6 years, and one last case occurred in 7 years and 9 month after the radiation therapy. Among 7 patients, 5 patients were treated with adjuvant radiation therapy after the surgery and 2 patients have received only curative radiation therapy. The radiation dose received varied between patients, ranging from 6000 cGy to 7400 cGy. The average dose of radiation a patient received was 6500 cGy. For example, patient with chordoma has received the different amounts of radiation (3000, 1600, and 6480 cGy) in each treatment visit for the first year. In addition, intraoral fistula occurred for all patients whereas extraoral fistula and pathologic fractures occurred to 5 patients. We also found that 6 patients had the history of tooth extraction at the site of osteoradionecrosis preoperatively or postoperatively (Figure 1). All patients were suffered from pain and trismus (Table 2). After the failure of conservative methods and when severe bone and soft-tissue necrosis prevailed, reconstruction using the fibular free flap was performed after the segmental mandible resection. Pull through approach and transoral approach were used for mandible resection. In two cases, resection was done at the site of mandible body only and for rest of cases, resection was performed in mandible ramus including coronoid process. Among 7 patients, only 1 patient was edentulous patient. After mandibular resection, segmental mandibulectomy with reconstruction using a fibular free flap was performed. For the patient who had a fistula, fistulectomy was done with a soft tissue graft in addition to fibular free flap (Figure 2). Tissue transplantation was successful for all the patients. Flap with a 4x12cm thickness was the most frequently used flaps for reconstruction and the bone, about 5–9 centimeters in length, was collected and used in the surgery (Figure 3). The ipsilateral neck vessels were most commonly used for anastomosis, except 1 patient who received anastomosis on the contralateral side as vessels in ipsilateral side were damaged by earlier radiation therapy treatment. Among the artery used for vascular anastomosis, superior thyroid artery was the most frequently used artery (6 times), facial artery was used twice, and transverse cervical artery was used once. For the veins used for anastomosis, both external jugular vein and facial vein were used 5 times each, and each internal jugular vein, transverse cervical vein and anterior jugular vein were used once (Table 3). Vein grafts were not used. Improvement in postoperative mouth opening was observed with 100% increase in range of mouth opening compared with the preoperative values (Figure 4). Furthermore, significant improvement was noted in relieving pain, treating trismus and chewing. The present study showed that the overall patient satisfaction was high (Table 4).

**Table 1:** **The baseline characteristics of patient and tumor profiles**

**Patient No.**	**Age (yr)**	**Sex**	**Location**	**Diagnosis**	**TNM stage**	**Previouse treatment**
1	52	M	Right gingiva mucosa	SCC	T2N0M0	RT alone 6500 cgy
2	52	F	Left gingiva mucosa	SCC	T2N0M0	OP + RT 7320 cgy
3	47	M	Lower lip	SCC	TXN2M0	OP + RT 6300 cgy
4	53	M	Infratemporal fossa	Chordroma		OP + RT 11480 cgy
5	64	F	Submandibular gland	MEC	T3N2cM1	RT alone 6000 cgy
6	55	M	Tongue	SCC	T2N0M0	OP + RT 7400 cgy
7	51	M	Left tongue	SCC	T2N1M0	OP + RT 6000 cgy

SCC, Squamous Cell Carcinoma; MEC, Mucoepidermoid Carcinoma; RT, Radiation Therapy; OP, Operation surgery.

**Table 2:** **Detail of postoperative outcomes**

**Patient no.**	**Pre-operation (n = 7)**	**Post-operation (n = 7)**
Fracture	5 (71)	0 (0)
Trismus	7 (100)	0 (0)
Extraction history	6 (85)	
Fistula		
Intraoral	7 (100)	0 (0)
Extraoral	5 (71)	0 (0)
Pain		
Severe	6 (85)	0 (0)
Moderate	1 (14)	0 (0)
Mild	0 (0)	1 (14)
No pain	0 (0)	6 (85)

Values are presented as number (%).

**Table 3:** **Mandibular reconstruction with free vascularized fibular flap**

**Patient no.**	**Skin paddle**	**Bone length**	**Artery**	**Vein**
1	4.0 × 12.0	5.0	Superior Thyroid	Facial and External jugular
2	3.0 × 8.0	5.0	Superior Thyroid	Facial and Internal jugular
3	4.0 × 12.0	5.0	Superior Thyroid	Facial and External jugular
4	4.0 × 12.0	6.0	Superior Thyroid	Facial and Anterior jugular
5	4.0 × 12.0	9.0	Superior Thyroid	Facial and External jugular
6	4.0 × 12.0	7.0	Superior Thyroid	Facial and External jugular
7	5.0 × 13.0	7.0	Transverse Cervical	Transverse cervical and External jugular

Values are presented as number (cm).

**Table 4:** **Maximum mouth opening limitation of pre-operation and post-operation**

**Patient no.**	**Pre-operation**	**Post-operation**
1	15.0	33.0
2	17.5	35.0
3	18.0	40.0
4	15.0	41.0
5	10.5	35.0
6	19.0	40.0
7	17.0	45.0

Values are presented as number (mm).

## Discussion

**Figure 5:**
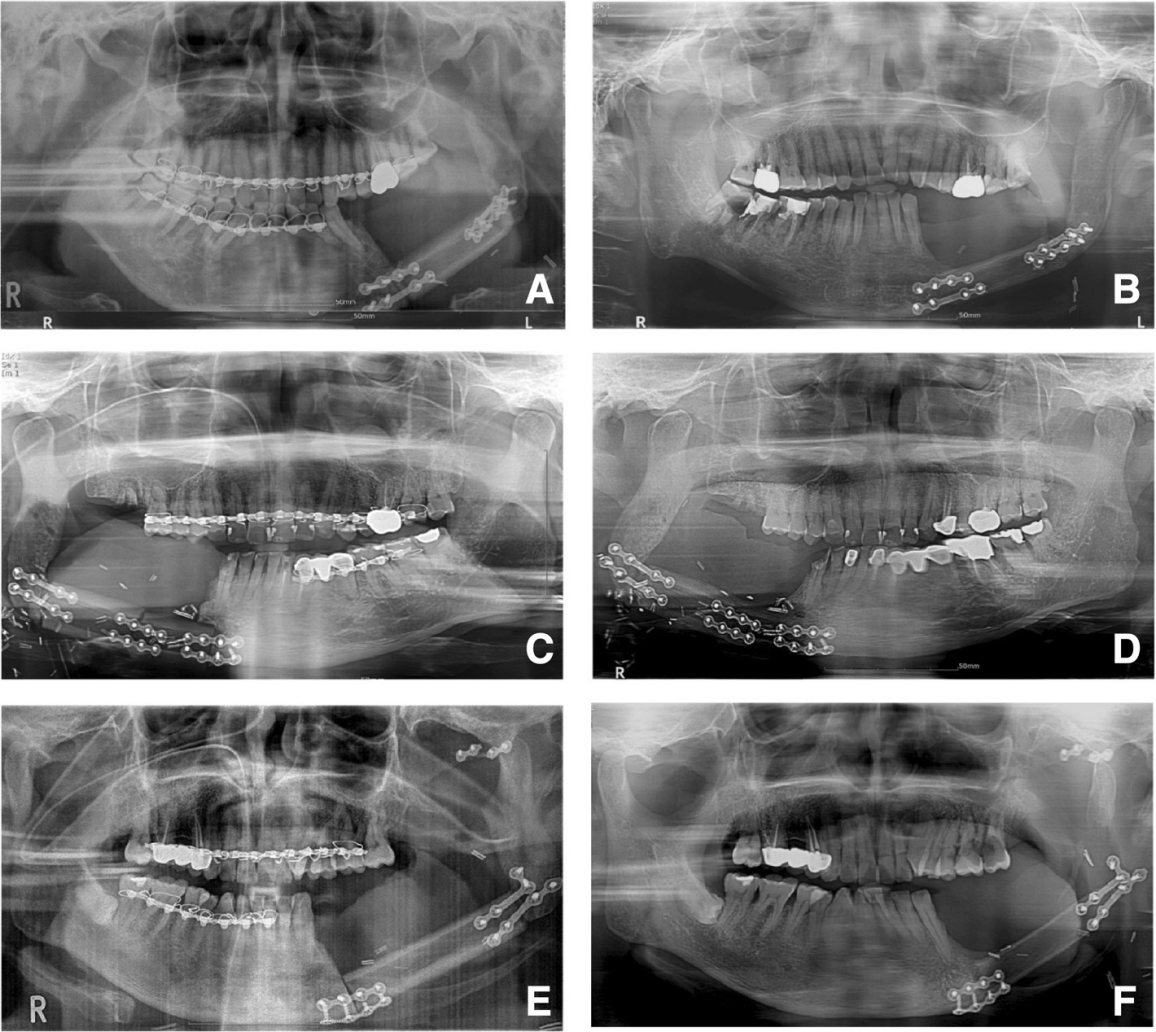
**Panoramic view of the postoperative fibular free flap follow ups.** Paired pictures refer to the same person; **(A)** – **(B)**, **(C)** – **(D)**, **(E)** – **(F)**. Pictures **(A)**, **(C)**, and **(E)** are postoperative panoramic view of two days after surgery. **(B)** and **(D)** shows panoramic view of miniplate fracture and bone resorption on the ramus and fibular distal area about 3 years after the surgery. **(F)** mandible ramus resorption is shown 5 years the after surgery. Trismus and midline deviation occurred.

Radiation therapy is an important treatment for oral cancer as it can be the primary treatment of curative therapy and also be used as part of adjuvant therapy. However, serious complications from radiation can occur not only in the cancer cell but also in the normal tissue around the cancer. Tissue density is an important factor to determine the radiation resorption. Bone is 1.8 times more organized than the soft tissue. Also, the maxilla is less dense than the mandible and osteoradionecrosis is more commonly observed in the mandible than in the maxilla. In this study, all patients had been diagnosed of osteoradionecrosis in the mandible. There are many different alternatives for osteoradioecrosis treatment and oseoradionecosis has been defined in many ways. Marx’s classification has been generally accepted method. Marx described osteoradionecrosis as a delay of advanced wound healing protocol due to the failure of wound healing response. According to Marx’s classification, there are 3 stages in osteoradionecorsis; stage 1, the presence of exposed alveolar bone without signs of pathologic fracture, which responds to hyperbaric oxygen (HBO) therapy; stage 2, disease does not respond to HBO, and requires sequestrectomy and saucerization; stage 3, involves full thickness bone damage or pathologic fracture, usually requires complete resection and reconstruction with free tissue [2-4]. Recently, the Notani classification by inferior alveolar canal in radiation picture is accepted to all cases of mandibular osteoradionecrosis. Class I refers to when osteoradionecrosis is limited to dentoalveolar bone. Class II refers to when necrosis progress above inferior alveolar canal. Class III is when oseteoradionecrosis progress to inferior alveolar canal or pathologic fracture occur [7]. Conservative treatments including antibiotic treatment, gargling or enhancing oral hygiene are the basic treatment for osteoradionecrosis. Various treatments have been used in osteoradionecrosis. However according to Happnen et al., 25 ~ 46% of patients have failed the long-term antibiotic treatment and had to receive mandibular resection due to the progression of necrosis [8]. In the study of Weissman and Rankow (1971), patients took a one-year non-surgical treatment such as antibiotic treatment and removal of the oral cavity stimulating factor, but 25% of failed patients still had to receive hemi-mandibulectomy [9]. In study of Drane and Daly, they repeated sequestrectomy to osteoradionecrosis patients and 64% of the patients had to receive segmental mandibulectomy [10]. Thereafter, ultrasonic, high-frequency electromagnetic stimulation and hyperbaric oxygen therapy which can accelerate the formation of new blood vessels and cells were introduced. Marx et al. have proposed a treatment protocol which is a basic requirement to use HBO therapy before and after the surgery combined with surgical debridement [2,4]. Hyperbaric oxygen therapy has been regarded as the effective treatment, but there is still ongoing debate about its effectiveness. Study by Annane et al. showed no benefit of hyperbaric oxygen therapy over placebo [11]. When conservative treatments are unsuccessful, surgical treatment is needed for the management of stage 2 and stage 3 osteoradionecrosis. Resection of wide range of tissue and reconstruction with free flap are the commonly suggested surgical intervention. Aggressive surgical approach is more effective when bone necrosis is advanced [6]. Fibular free flap provide support for dental implantation and denture which helps in recovery of occlusal function. Since a donor site is away from a receiving site, 2 team approaches are available. It is known to have a very high success rate and useful in the reconstruction of mandible. In addition, fibular free flap can provide sufficient amount of bone and soft tissue for mandible reconstruction with a minor risk of donor site complications [12]. Blood vessels harvesting procedure is required for fibular free flap to suppy the tissue at the recipient site and an appropriate length of the blood vessles must be selected to provide anastomosis with no tension. However, the recipient vessels may be available for anastomosis due to a prior neck dissection surgery and a prior radiation therapy. Vein grafts are occasionally required under certain circumstances. Vein grafts from the saphenous vein is considered to be the most versatile and reliable vein graft for an interposition. However, vein graft is known for high risk of thrombosis and hemorrhagic complications. Vein graft should be used carefully [13]. This study demonstrates that fibula free flap is a safe and reliable method for comprehensive functional and esthetic mandibular defect reconstruction. All patients showed the good functional results related to the pain, trismus and chewing. In the study of Lin Wang et al., mandible reconstruction with fibula free flap effectively eliminated pain and trismus, whereas there were no significant improvement of swallow, speech and xerostomia [14]. Our study showed similar result to Lin Wang et al. Furthermore, improvement in occlusal function can be expected through the dental implant installation. Through the long term follow up study, we observed the panoramic view of the miniplate fracture and the bone loss in the mandible ramus and fibular bone junction (Figure 5). In addition, patients showed trismus and incisor deviation. All patients had the occlusal function through non-surgical side of teeth. Since occlusal load was concentrated in the opposite site of teeth after the surgery, stress was concentrated in the ramus fibular bone junction which generated bone resorption and miniplate fracture [15]. To overcome this stress barring effect, more stress shielding methods in the mandible ramus and fibula junction were required, such as increasing miniplate unit. However, further studies are needed on this topic.

## Conclusions

The mandible reconstruction with fibula free flap is a stable method which effectively eliminates pain and trismus. We can also expect the improvement of chewing and swallowing by dental implantation in the reconstruction site. However, there are remaining limitations. A long period of time is required for osteoradionecrosis treatment. Also, there are serious complications of osteoradionecrosis remaining such as severe facial deformity, intolerable pain, loss of occlusal function and fistula. It is difficult to say that the treatment of oral cancer was successful if oral malfunction related to osteoradionecrosis still remains. It is important to keep a long term follow up study as recurrence of osteoradionecrosis can happen. Excellent oral hygiene care and minimizing dental trauma can reduce the risk of osteoradionecrosis.

## Consent

Written informed consent was obtained from the patient for the publication of this report and ant accompanying images.
